# Selective and mild fractionation of microalgal proteins and pigments using aqueous two‐phase systems

**DOI:** 10.1002/jctb.5711

**Published:** 2018-07-03

**Authors:** Catalina A Suarez Ruiz, Daniel P Emmery, Rene H Wijffels, Michel HM Eppink, Corjan van den Berg

**Affiliations:** ^1^ Bioprocess Engineering, AlgaePARC Wageningen University Wageningen The Netherlands; ^2^ Faculty of Biosciences and Aquaculture Nord University Bodø Norway

**Keywords:** microalgae biorefinery, proteins, pigments, cholinium‐based ionic liquids, aqueous two‐phase systems

## Abstract

**BACKGROUND:**

Microalgal biomass is generally used to produce a single product instead of valorizing all of the cellular components. The biomass production and downstream processes are too expensive if only one product is valorized. A new approach was proposed for the simultaneous and selective partitioning of pigments and proteins from disrupted *Neochloris oleoabundans* cultivated under saline and freshwater conditions.

**RESULTS:**

An aqueous two‐phase system composed of polyethylene glycol and cholinium dihydrogen phosphate selectively separated microalgal pigments from microalgal proteins. 97.3 ± 1.0% of lutein and 51.6 ± 2.3% of chlorophyll were recovered in the polymer‐rich phase. Simultaneously, up to 92.2 ± 2.0% of proteins were recovered in a third phase (interface) in between the aqueous phases (interface). The recovered proteins, including Rubisco with a molecular weight of ∼560 kDa, seem to be intact and pigments did not suffer degradation, demonstrating the mildness of this system for fractionating microalgal biomolecules.

**CONCLUSION:**

The ability of aqueous two‐phase systems (ATPSs) to simultaneously and efficiently fractionate different biomolecules in a mild manner from disrupted microalgae is demonstrated. This is an important step towards the development of a multiproduct microalgae biorefinery. © 2018 The Authors. *Journal of Chemical Technology & Biotechnology* published by John Wiley & Sons Ltd on behalf of Society of Chemical Industry.

## INTRODUCTION

Microalgae are a promising feedstock for many industries including biopharmaceuticals, biomaterials, bioenergy, nutraceuticals, agriculture, animal health and cosmetics and personal care.^1^ Feasibility studies demonstrate that a biorefinery focus on one single product is not cost‐efficient^2^ and that several microalgal components should be extracted to supply different markets, and thus increase the overall value of microalgal biomass.^3^

Depending on the strain, microalgae can contain chlorophylls (green), carotenoids (red, orange and yellow) and phycobiliproteins (red and blue).^4^, ^5^ *Neochloris oleoabundans* accumulates mainly lutein, cantaxanthin, zeaxanthin, and astaxanthin monoesters and diesters, which are promising ingredients for pharmaceutical and nutraceutical applications.^6^, ^7^ Moreover, microalgal proteins are recognized for their high quality, showing outstanding nutritional, functional and techno‐functional properties that are in some cases superior to conventional protein concentrates.^8^, ^9^

Although microalgal cells contain several valuable biomolecules, the currently applied separation processes valorize only one specific product (e.g. astaxanthin, phycobiliproteins).^10^, ^11^ The development of efficient, mild and scalable methods/processes, capable of fractionating different microalgal biomolecules, is of particular importance.^12^ For the fractionation of hydrophobic molecules such as lipids and pigments from disrupted microalgae, organic solvents are often used, whereas water‐soluble components such as proteins and carbohydrates are discarded or undervalued.^13^ Proteins are fragile molecules that tend to denature during extraction using organic solvents or other harsh conditions.^14^ For a complete valorization of microalgal biomass, it is favourable to use mild separation methods.

Aqueous two‐phase systems (ATPSs) have been adopted as a new technology in a microalgae biorefinery framework. An ATPS is composed of two immiscible aqueous phases formed generally by two polymers, a polymer and a salt or two salts. Because an ATPS contains mainly water, the phases formed can provide a mild and suitable environment for biomolecules.^15^ Furthermore, the process of ATPS extraction is easy to scale up, uses up nonflammable/volatile and low toxic components, and simultaneously separates multiple products.^16^

The potential for the refinery of biomolecules by using ionic liquids (IL)‐based ATPSs (IL‐ATPSs) has grown exponentially.^17^ IL‐ATPSs provide advantages over conventional ATPSs because: they are highly tuneable through variations in pH, molecular structure, composition and temperature; and the wide variety of potential ions spans the entire hydrophobicity–hydrophilicity range.^18^ Thus, IL‐ATPSs potentially have a higher selectivity, flexibility and can provide more efficient separations than commonly used ATPSs composed of polymer‐polymer or polymer‐salt.^14^ However, reports on integrated separation processes of biomolecules from biomass using ATPSs and IL‐ATPSs are limited.^19^, ^20^, ^21^, ^22^ The application of IL‐ATPSs is generally studied using pure biomolecules (e.g. proteins), facilitating the understanding of the partitioning behaviour of certain molecules. However, these compounds are normally present in complex biological matrices such as microalgae biomass, which contain a large number of other components (e.g. carbohydrates, pigments, lipids). Therefore, to provide a realistic scenario, investigations using IL‐ATPSs need to focus on the fractionation of these compounds from complex matrices.^23^

Studies of ATPSs for the fractionation of biomolecules from microalgae extracts are limited. These studies mainly focus on the recovery of specific molecules such as the fluorescent pigment‐protein complex *C‐phycocyanin* from *Spirulina* strains,^24^ *B*‐phycoerythrin from *Porphyridium cruentum* 25, 26 and proteins from *Chlorella sorokiniana*.^27^ The simultaneous separation of different molecules from microalgae has not been addressed.^28^ To our knowledge, the current study is the first study using IL‐ATPSs for the simultaneous fractionation of proteins and pigments from disrupted microalgae.

In this research, *Neochloris oleoabundans* is used to study the fractionation of pigments and proteins by using one conventional ATPS (polymer/salt) and two IL‐ATPSs. Mild and biocompatible phase‐forming components were selected based on previous investigations.^29^ ATPS was used for the fractionation of pigments and proteins from disrupted *N. oleoabundans* grown under both freshwater and saline conditions.

## MATERIALS AND METHODS

### Chemicals

Potassium citrate tribasic monohydrate, polyethylene glycol (PEG) 400, hydrochloric acid, acetic acid and Bovine serum albumin protein (BSA), >98% were purchased from Sigma‐Aldrich (Zwijndrecht, The Netherlands). Citric acid was obtained from Merck. The ILs, IoliLyte 221 PG, >95% and cholinium dihydrogen phosphate (Ch DHp), >98%, were obtained from Iolitec. Acetonitrile and Methanol (HPLC grade) were provided by Biosolve and ethyl acetate by Fisher Scientific.

### Microalgae cultivation, harvesting and cell disruption

*Neochloris oleoabundans* (UTEX 1185, University of Texas Culture collection of Algae, USA) was cultivated in a fully automated 1300 L vertical stacked tubular photo bioreactor (PBR) located at AlgaePARC, The Netherlands. It was cultivated under saline and freshwater conditions, both in Bold's Basal medium^30^ at a pH value of 8.0 and the temperature was controlled at 30 °C. To cultivate microalgae under saline conditions artificial seawater was used: NaCl: 24.5 g L^−1^; MgCl_2_: 9.8 g L^−1^; CaCl2: 0.53 g L^−1^; K_2_SO_4_: 0.85 g L^−1^; NaSO_4_: 3.2 g L^−1^; NAHCO_3_: 0.8 g L^−1^. The microalgae were harvested (80 Hz, 3000 × g, 0.75 m^3^ h^−1^) using a spiral plate centrifuge (Evodos 10, Evodos, The Netherlands). The biomass paste was suspended in MilliQ® water to obtain a solution containing 6% of solids and was disrupted by using a horizontal stirred bead mill (Dyno‐Mill ECM‐AP) using zirconia beads with bead size of 0.5 mm. Bead‐milled microalgae was centrifuged (20 min, 20 000×*g*) to separate most of the cell debris from the supernatant. The resulting extract (supernatant) was stored at –20 °C until further use.

### Fractionation of pigments and proteins from microalgae using ATPSs

Previously, three promising ATPSs were selected and characterized for the separation of microalgae components.^29^ The selection of the ILs was based on their interaction with the protein Ribulose‐1,5‐biphosphate carboxylase/oxygenase (Rubisco), a protein present in microalgae with a molecular mass of ∼560 kDa consisting of noncovalently bound eight large subunits (∼56 kDa) and eight small subunits (∼14 kDa). Potassium citrate and PEG 400 were selected due to their biocompatibility and ability to form ATPS with these ILs. The composition of the mixtures prepared for the partitioning of microalgal pigments and proteins is shown in the Table 1S of the supporting information.

The ATPS mixtures were prepared gravimetrically (10^−4^ g) by using mixture points along four tie‐lines (TLs) with a volume ratio (*V*_r_) of one between the top and bottom phase as described by Suarez Ruiz *et al*.^29^ One gram of microalgal extract was added and MilliQ® water was used to complete 10 g in each system. Mixtures were protected from light during the fractionation process. These were stirred for 1 h in a rotatory shaker (50 rpm) and left to equilibrate at room temperature. To facilitate the separation of the aqueous phases from the interface all systems were centrifuged for 5 min at 1200×*g*. The phases (top, bottom and interface) were carefully separated and the volume and weight were noted. The interfaces were resuspended in MilliQ® water to facilitate the quantification of the biomolecules. Possible interferences of phase‐forming components on the analytical method were taken into account, and control samples were prepared using water instead of microalgae extract. At least two individual samples for each condition were prepared and the biomolecules were quantified. The results were reported as the average of two independent experiments with the respective standard deviation.

### Pigment analysis

Pigments in each phase (top, bottom and interface) were analysed by measuring the absorption spectrum between 200 and 750 nm. Total chloroplast carotenoids and chlorophylls were determined by measuring their absorbance at 470 nm using a spectrophotometer (DR6000, Hach Lang, USA). Relative partition coefficients (*K*_p_) for total pigments were calculated by Eqn (1). Pigments from the initial microalgal extract and interfaces after ATPS were completely extracted with methanol as a control for further calculation.

(1) $$Kp_{\text{Pigment}s}=\frac{A_{470\mathrm{nm},\mathrm{top}}}{A_{470\mathrm{nm},\text{bottom}}}$$

### Reversed‐phase high‐performance liquid chromatography

RP‐HPLC was used to identify and quantify the pigments after separation in ATPS. HPLC analysis was performed in a Shimadzu system coupled with a photo‐diode array detector (SPD‐M20A) and an Acclaim™ C30 LC reversed‐phase column from Thermo Scientific™ was used. Three mobile phases were used: (A) Acetonitrile, (B) Methanol/Ethyl acetate 1:1 (v/v) and (C) 200 mmol L^–1^ Acetic acid in water with the gradient shown in Table 1. The flow rate was set at 1.5 mL min^−1^ and the column temperature at 30 °C.

Three pigments were identified and quantified (lutein, chlorophyll a, chlorophyll b) and extraction efficiencies (%w/w) were calculated with Eqn (2), where *m*_*Lutein*, *initial*_ is the initial mass of lutein in the microalgae.

(2) $${\mathrm{EE}}_{\text{Lutein}}\%=\frac{C_{\text{Lutein},\mathrm{top}}*V_{\mathrm{top}}}{m_{\text{Lutein},\text{initial}}}$$

**Table 1:** HPLC gradient method

Time (min)	%A	%B	%C
0	85.0	14.5	0.5
2	85.0	14.5	0.5
15	65.0	34.5	0.5
25	65.0	34.5	0.5
30	85.0	14.5	0.5

### Protein analysis

Proteins in each phase were separated from the phase forming components and quantified by Size Exclusion Chromatography (SEC) on an Äkta pure FPLC system equipped with a Hi Trap 5 mL desalting column with Sephadex G‐25 resin (both GE Healthcare), inline detectors and a fraction collector. Samples were injected using a 100 µL injection loop system and were eluted with 0.05 mol L^–1^ sodium phosphate, 0.15 mol L^–1^ sodium chloride pH 7 buffer at a flow rate of 1 mL min^−1^ at room temperature. Peak detection was conducted by a UV/Vis absorbance detector at a wavelength of 280 nm and to control the purification process conductivity and pH monitors were used. For protein quantification, FPLC chromatograms at 280 nm were integrated using the GE Unicorn software. Samples were collected after purification by FPLC and protein content was determined by Bradford's method^31^ using the Pierce™ Coomassie Plus (Bradford) Assay Kit. Absorbance at 595 nm was measured using a Tecan infinite M200® plate reader. Calibration curves were prepared for both quantification methods with Bovine (BSA) pure protein in MilliQ® water.

Partition coefficient (*K*_p_) was calculated from the ratio of protein concentrations between top and bottom phase [Eqn (3))]. Protein distribution between the three phases was described by the protein extraction efficiency in each phase (***EE***_***Protein***_%) which expresses the ratio of protein amount between the top, interface or bottom phase and the total amount [Eqn (4))]. ***m***_***protein*,*initial***_ is the initial mass of protein in the microalgae extract added.

(3) $$K_{p\;\text{protein}}=\frac{C_{\text{protein},\text{top}}}{C_{\text{protein},\text{bottom}}}$$

(4) $${\mathrm{EE}}_{\text{Protein}}\%=\frac{C_{\text{protein},\mathrm{top}}*V_{\mathrm{top}}}{m_{\text{protein},\text{initial}}}$$

The selectivity of pigments considering the presence of proteins in the top phase was calculated with Eqn (5):

(5) $$S_{\text{pigment}/\text{protein}}=\frac{K_{p\;\text{pigments}}}{K_{p\;\text{proteins}}}$$

### Statistical analysis

All experiments were conducted in duplicate and results were reported as the average of two independent experiments with the respective standard deviation. Statistical analysis was performed using Statistica 10.0 software. One‐way ANOVA and Tukey honestly significant difference tests were implemented to assess significant differences among the different treatments.

### Electrophoresis

In order to investigate the conformation of the proteins before and after the partitioning step, the samples were analysed by native gel electrophoresis (Native‐PAGE). The samples were diluted with native sample buffer in the ratio 1:2 and applied on a 4–20% Criterion TGX (Tris‐Glycine eXtended) precast gel. The gel was run in a 10× Tris glycine native buffer at 125 V for 75 min. The native gel was stained with the Pierce Silver Stain Kit from Thermo Scientific. The precast gels and buffers were procured from Bio‐Rad.

## RESULTS AND DISCUSSION

**Figure 1:**
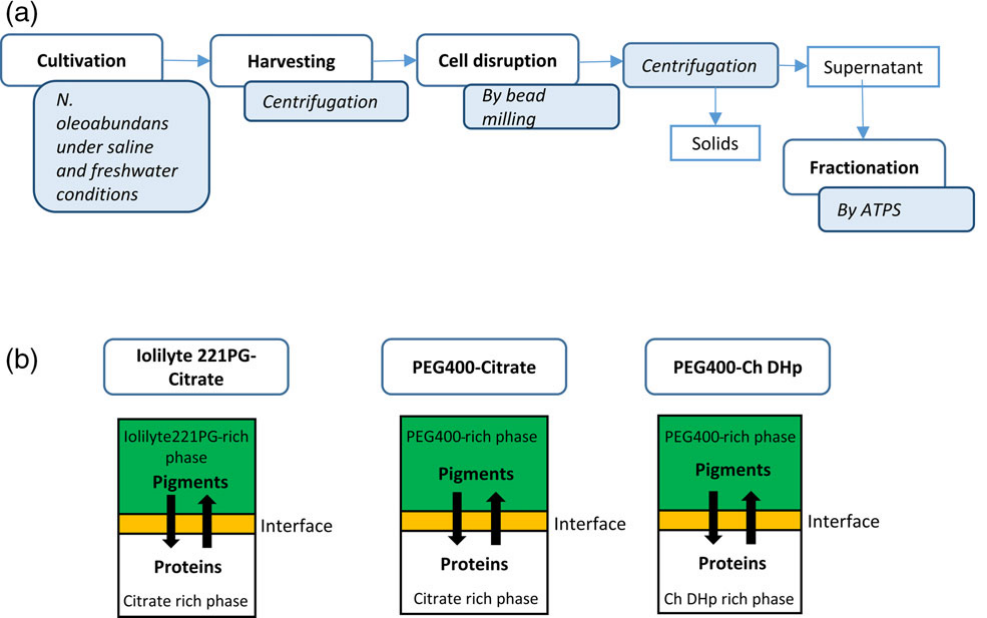
(a) Brief diagram of the process followed in the separation of proteins and pigments from microalgae; (b) Description of the main phase‐forming components in each aqueous two‐phase system (ATPS).

Three ATPSs – polyethylene glycol (PEG) 400‐potassium citrate, Iolilyte 221PG‐potassium citrate and PEG400‐Cholinium dihydrogen phosphate (Ch DHp) – were selected to evaluate the partitioning of pigments and proteins from *N. oleoabundans* extract. These systems were selected previously based on their interaction with the protein Rubisco as explained by Suarez Ruiz *et al*.^29^ Microalgae cultivated under saline and freshwater conditions were used to investigate the feasibility of this technique in a multiproduct biorefinery approach. A brief schematic representation of the process presented in this paper is shown in Fig. 1(a).

### Pigment partitioning in ATPSs

**Figure 2:**
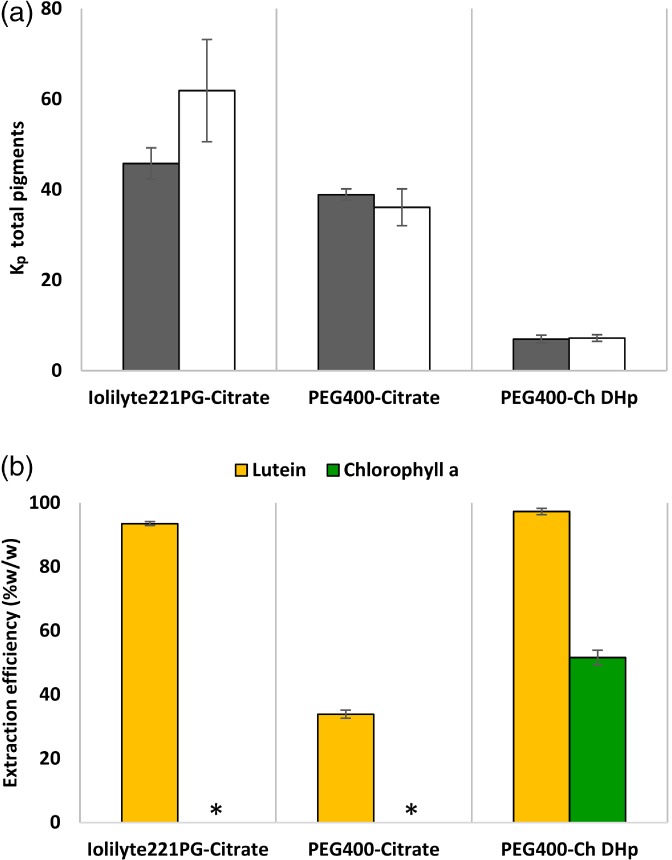
Pigment separation from *N. oleoabundans* extract in ATPSs. (a) Partition coefficient (*K*_p_) for total pigments. Saline (filled bars) and freshwater (open bars) cultivation conditions for *N. oleoabundans*. (b) Extraction efficiencies (%w/w) in the top phase for lutein and chlorophyll a from microalgae cultivated in saline water. The results represent the average of two independent experiments and error bars indicate standard deviations. Asterisks (*) denote chlorophyll a not detected by the RP‐HPLC method.

As described in Fig. 1(b), Iolilyte 221PG‐citrate consists of a top phase rich in IL (Iolilyte 221PG) and a bottom phase rich in citrate. PEG400‐citrate and PEG400‐Ch DHp ATPSs both consist of a top phase rich in polymer (PEG400) and a bottom phase rich in potassium citrate and Ch DHp, respectively. *Neochloris oleoabundans* pigments (carotenoids and chlorophylls) are hydrophobic molecules and therefore tend to partition to the least‐hydrated phase (top phase) as shown by the partition coefficient values (*K*_p_ > 1) in Fig. 2(a). The highest partition coefficients were obtained with Iolilyte221PG‐citrate ATPS (*K*_p_ = 62 ± 11), followed by PEG 400‐citrate (*K*_p_ = 36 ± 4) and PEG 400‐Ch DHp (*K*_p_ = 7 ± 0.7). Partition coefficient values and extraction efficiencies presented in Fig. 2 were obtained using mixture points along the highest tie line lengths (TLLs). Additionally, in the supporting information, Table 1S provides the concentration of phase‐forming components for each TLL and Table 2S provides the effect of the TLL on the *K*_p_ of total pigments.

The effect of the phase composition was investigated based on the TLL, which represents the composition and thermodynamic difference of the two phases. The partition coefficient of pigments tends to increase with the TLL and this effect seems to be consistent in the three systems (Table 2S). Increasing the component concentration in the three systems will enhance the salting out effect, resulting in a less hydrated top phase and an increase of the pigments concentrated in this phase.

Few authors have studied the partitioning of pigments and carotenoids in ATPSs and they all show that the recovery of these highly hydrophobic molecules is higher when the hydrophobic nature of the top phase is increased.^20^, ^32^, ^33^ Montalvo‐Hernández *et al*.^34^ studied the partitioning of crocins (carotenoids) in four types of ATPSs: polymer–polymer, polymer–salt, alcohol–salt and IL–salt. In the polymer–salt system, increasing molecular weight and TLL leads to higher recovery of the carotenoids due to the higher hydrophobicity of the upper phase. Similarly, for IL–salt, hydrophobic interactions related to the alkyl chain length of the cation seem to be the main driving force for the partitioning of this carotenoid. PEG molecular weight is directly related to PEG hydrophobicity. PEG and Iolilyte 221PG were compared previously and the higher ability of Iolylite 221PG to form ATPS is related to its higher hydrophobicity nature as a consequence of its alkyl chain length and molecular weight.^29^ Comparing the molecular weight of the polymer and the IL, Iolilyte 221PG (*n* = 5–15) is a more hydrophobic molecule in comparison with PEG 400 (*n* = 9) (Table 2). This could explain the higher recovery of total pigments in Iolilyte221PG‐citrate.

**Table 2:** Molecular structure of two important pigments in *N. oleoabundans* and the main components of the top phases

Molecule	Chemical structure
Lutein	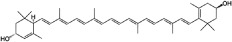
Chlorophyll a	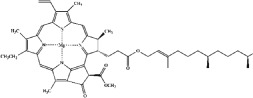
Polyethylene glycol 400 (PEG 400)	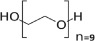
Iolilyte 221PG	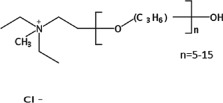

The hydration of the bottom phase main component also is important in the separation of hydrophobic molecules.^33^ In the case of PEG400‐Ch DHp, the high affinity of the IL for water may lead to a higher partitioning of pigments to the top phase^33^, ^35^. However, interactions between the polymer and the IL play an important role in the partitioning of biomolecules, making the partitioning of the molecules more difficult to predict.^36^ Nevertheless, the extraction of hydrophobic molecules in polymer–IL ATPSs could be enhanced by the correct selection of the IL employed based on these observations.

The salinity of the medium did not affect the preference of the pigments to migrate towards the most hydrophobic phase (*K*_p_ > 1). Microalgae cultivated under saline and freshwater conditions were used to evaluate the feasibility of using ATPSs to fractionate biomolecules from different sources (e.g. microalgae cultivated under different conditions). No significant differences were found (*P* > 0.05) when comparing the partition coefficients of total pigments from microalgae cultivated under saline and freshwater conditions. This is in agreement with the *K*_p_ results obtained increasing the concentration of NaCl% in the ATPS mixtures. Figure 1S (supporting information) presents the effect of salt concentration (NaCl, %w/w) on the partitioning of pigments. Although a slight increase trend is observed, only the *K*_p_ obtained with 3% NaCl in PEG 400‐Ch DHp was significantly higher (*P* < 0.05). The medium to cultivate microalgae under saline conditions was prepared using 2.5 (%w/w) of NaCl. Thus, it does not seem that the salinity in the cultivation medium affects the partitioning of pigments. This is an advantage for the future application of the process, because no washing steps would be necessary before the fractionation process.

Relative partition coefficients were calculated based on the absorbance of total pigments using UV–Vis spectrophotometry at 470 nm. UV‐Spectroscopy is used widely to analyse the absorption spectrum of pigments in different solvents^37^. Although it provided information about the overall pigment spectrum, the quantification of specific pigments (e.g. chlorophyll a) was affected by their degradation products. The absorption spectrum of the degradation products of pigments overlapped with the target pigments (e.g. chlorophyll a).^38^, ^39^ Therefore, the quantification of specific chlorophylls and carotenoids (lutein, chlorophyll a and chlorophyll b) was done by HPLC. This method allowed us to identify the individual pigments of interest in the microalgae and to calculate the recovery of these pigments in the top phase. Chromatograms of the pigments present in microalgae under saline conditions and recovered in the top phase using ATPSs are shown in Fig. 2S. This figure shows that lutein had the highest peak followed by chlorophyll b and chlorophyll a (quantified at 660 nm). Lutein was efficiently recovered by Iolilyte 221PG‐citrate (93.1 ± 0.6%) and PEG400‐Ch DHp (97.3 ± 1.0%), whereas PEG400‐citrate only recovered 34.8 ± 1.26%. Chlorophyll a was recovered by PEG400‐Ch DHp (50.6 ± 2.3%), but it was not detected in the other two systems at 660 nm [Fig. 2(b)]. Exceptionally, chlorophyll b was not found in any of the top phases. The lack of chlorophyll a and chlorophyll b in some ATPSs could be due to low extraction efficiencies or a change in the molecule structure by oxidation. Chlorophyll stability is affected by temperature, light irradiance, acids, bases and oxygen, causing the loss of its magnesium ion and/or phytol group^40^ and a change in colour to olive‐brown.^41^ Pigments were not detected in the interfaces and bottom phases by RP‐HPLC, due to the lack of or insufficient amount of pigments present in those phases.

The nature, stability and amount of the pigments extracted by ATPSs depend on the phase‐forming components. It was observed that the green colour of the top phases was different in each case, being more brownish for Iolilyte‐221PG and PEG400‐citrate than for PEG‐Ch DHp. The brown colour might indicate modification of chlorophyll caused by oxidation, which is in agreement with former studies,^42^ where it was established that the quality of chlorophyll could be reduced by some ILs such as propylammonium nitrate yet enhanced by others such as dimethylethylammonium methanoate.

### Protein partitioning in ATPSs

**Figure 3:**
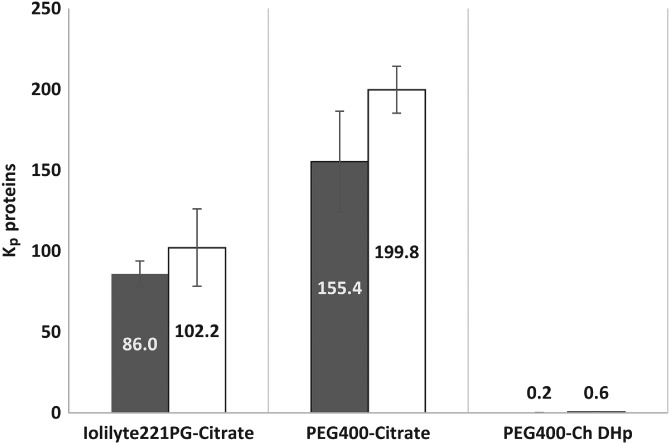
Protein partition in ATPS. Partition coefficient (*K*_p_) values for proteins using three different ATPSs. Saline (filled bars) and freshwater (open bars) cultivation conditions for *N. oleoabundans*. The results represent the average of two independent experiments and error bars indicate standard deviations.

Proteins were analysed in the phases after fractionation with ATPSs. Figure 3. shows the partition coefficient of proteins in the three systems using saline‐ and freshwater‐cultivated *N. oleoabundans*. The *K*_p_ values show large differences between PEG400‐citrate and Iolilyte 221PG‐citrate, in which proteins migrate preferentially to the top phase (high *K*_p_ values), and PEG400/Ch DHp, in which *K*_p_ values are below 1, showing a preference of the proteins to the opposite phase.

**Figure 4:**
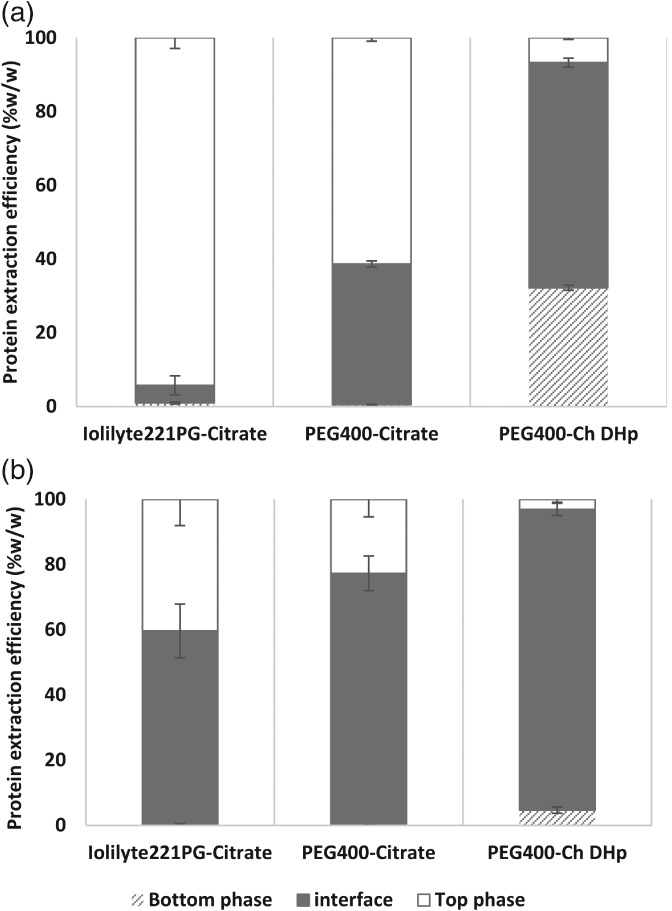
Distribution of microalgae proteins among the three phases in the three ATPSs. (a) Saline and (b) freshwater cultivation condition of *N. oleoabundans*. The results represent the average of two independent experiments and error bars indicate standard deviations.

The distribution of the proteins among the three phases is shown in Fig. 4(a) using *N. oleoabundans* cultivated under saline conditions and Fig. 4(b) using *N. oleoabundans* cultivated in freshwater. Extraction efficiencies of proteins from microalgae cultivated under saline conditions [Fig. 4(a)] into the top phase was 94.3 ± 2.8% using Iolilyte 221PG‐citrate and 61.3 ± 0.9% using PEG400‐citrate. These extraction efficiencies (%w/w) are eight to 13 times higher than that found using PEG400‐Ch DHp (6.7 ± 0.5%). In previous studies using Rubisco as model protein, results suggested that the high recovery of protein into the IL‐rich phase (Iolilyte 221PG) compared to polymer‐based ATPSs is a result of hydrophobic and electrostatic interaction between the phase‐forming components and the protein.^29^ This conclusion is in line with our current results. However, despite the high protein recoveries obtained with Iolilyte 221PG‐citrate, the pigments also are recovered in the top phase. As the pigments also are recovered in the top phase, this does not result in the multiproduct biorefinery we are aiming for. Furthermore, a third phase was formed increasing the complexity of the phenomena behind the partitioning of the molecules.

Aqueous two‐phase systems can generate interfacial partitioning of different molecules, including proteins, and it has gained attention in the last years for large scale‐processes.^43^ In the traditional ATPS (polymer–salt), protein partitioning is governed by hydrophobic interactions and the salting out effect. Similarly, the interfacial concentration of proteins in polymer–salt ATPSs was described by Kim^44^ based on a protein solubility model, in which protein precipitation was a result of increasing the salt concentration in the bottom phase. Thus, salting out was considered to be the most important driving force together with polymer‐excluded volume to concentrate proteins in the interface. In conventional ATPSs, polymer steric exclusion effects and hydrophobic interactions between the polymer and proteins often were mentioned to predict the maximum concentration that can be added to separate proteins into the top phase.^45^, ^46^ Protein precipitation is caused by phase saturation, determined mainly by hydrophobic interactions and by the salting out effect. However, the solubility of proteins seem to be different in each ATPS,^47^ due to the different partitioning driving forces involved.

Ionic liquid‐based three phase partitioning (ILTPP) has been investigated because it combines the advantages of the IL‐ATPS and TPP for the concentration and recovery of different molecules including proteins.^48^, ^49^, ^50^ ILTPP is capable of inducing the formation of a dense and stable protein layer in the middle with IL‐ATPS phase‐forming components. Alvarez‐Guerra and Irabien^51^ suggested that the salt concentration has the greatest influence on the amount of lactoferrin recovered at the interface. However, pH, temperature and protein concentration also influence the partitioning.^52^

The three systems studied in the current research were used previously for the partitioning of different proteins, including Rubisco.^14^, ^29^, ^35^ No precipitation of these proteins was reported, demonstrating that the studied systems do not always form three phases. Protein partitioning was studied previously using a total concentration of 0.3 mg mL^−1^ of purified Rubisco. In the current study similar microalgal protein amounts were used; 0.4 mg mL^−1^ for freshwater‐cultivated *N. oleoabundans* and 0.2 mg mL^−1^ for *N. oleoabundans* cultivated under saline conditions. However, the disrupted microalgae added to the systems contained other biomolecules, such as pigments, lipids, carbohydrates and other proteins apart from Rubisco. The overall composition of microalgae is influenced by cultivation conditions.^53^ Microalgae cultivated in freshwater has higher amount of proteins and pigments than microalgae cultivated under saline conditions. When using *N. oleoabundans* cultivated in freshwater more proteins were concentrated in the interface [Fig. 4(b)] than when using *N. oleoabundans* cultivated under saline conditions. This increase reflects a lower solubilizing capacity of the ATPSs on microalgae molecules (e.g. proteins) in these conditions, which may be caused by protein content but also by other molecules present in the microalgae. PEG400‐Ch DHp recovered 92.2 ± 1.9% of proteins in the interface, followed by PEG400‐citrate (77.2 ± 5.3%) and Iolilyte 221PG‐citrate system (59.2 ± 8.2%). We hypothesized that the interfacial precipitation can be caused by the amount of feedstock (including microalgae proteins) added and by the presence of other biomolecules from the microalgae that benefit the emulsification and precipitation of the proteins.

Other authors have reported that ATPSs may form an interface when high protein concentrations are added to the system as well as by increasing the TLL.^54^, ^55^, ^56^ In Table 3S, the distribution of proteins among the three phases and the influence of the TLL were reported. By increasing the TLL (phase‐forming components concentration), more proteins were recovered in the interface instead of the top phase. Temperature, polymer molecular weight, TLL and protein loading affect the solubilizing capacity of an ATPS.^47^ In a descriptive model of interfacial partitioning in an ATPS (polymer–salt), Luechau *et al*.^43^ suggested that the interfacial concentration of molecules depends on the phase system, feedstock composition and bioparticles at the interface. Particle size and interfacial tension of the system were used to describe the adsorption of particles in the interface.

In the PEG400‐Ch DHp system, proteins were concentrated mainly in the interface and in the bottom phase when using both cultivation conditions. Previous studies have reported the preference of pure proteins to the bottom phase when using ATPS combinations with a more hydrophobic polymer (PPG400) and cholinium‐based ILs.^35^, ^57^ Li *et al*.^57^ studied protein partitioning using systems composed of PPG 400 and various cholinium based‐ILs. They reported a decrease in the protein extraction efficiency in the IL‐rich phase due to an increase in protein size. The proteins studied were Lysozyme, Papain, Trypsin and BSA. They argued that protein partitioning to the IL‐rich phase (bottom phase) requires energy to break the interactions between phase components. Thus, smaller proteins require less energy than larger proteins to migrate into the bottom phase. Previous results reported by us agree with this theory.^29^ Rubisco with a higher molecular weight (∼560 kDa) than the proteins reported by Li *et al*.^57^ was partitioned preferentially to the PEG‐rich phase (top phase) when using PEG400‐Ch DHp. This indicates the importance of protein size in this particular ATPS formed by polymer and cholinium based‐ILs. Other studies explained the effect of protein structure (e.g. surface amino acid residues) on the partitioning of these molecules using ATPSs formed by polymer and ILs.^35^, ^58^ Concluding that protein partitioning depends on specific interactions (e.g. electrostatic) between the protein and the IL.

Microalgae contain a high amount of proteins with different molecular weights and structures, making the prediction of protein partitioning in PEG400‐Ch Dhp even more difficult. Molecular interactions between the proteins and the IL (Ch DHp) seems to be a strong driving force for the partitioning of proteins to the bottom phase. However, protein size, feedstock load and the presence of other molecules could trigger the interfacial partitioning of the proteins.

**Figure 5:**
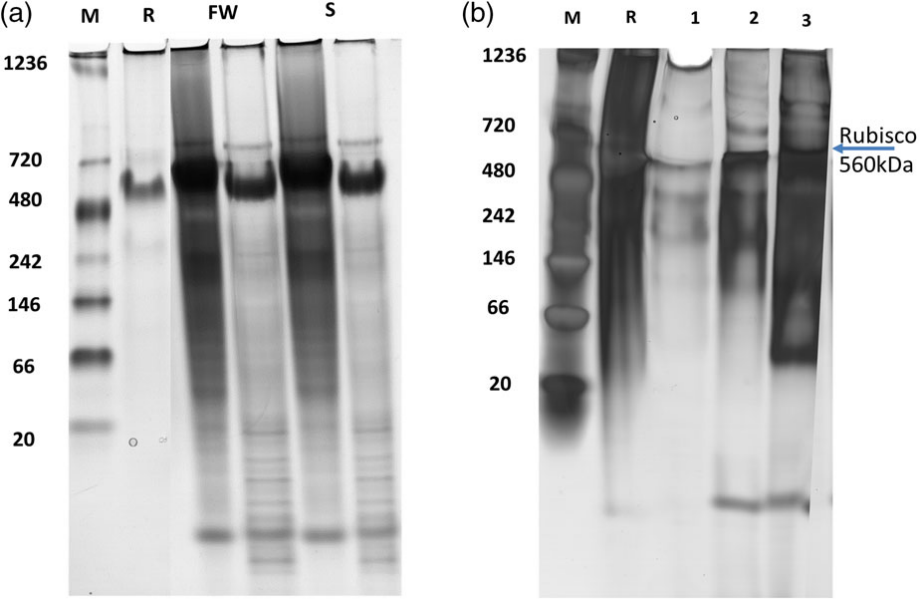
Protein conformation by Native PAGE: M, marker; R, Standard Rubisco (a) Proteins in *N. oleoabundans* cultivated in fresh water (FW) and saline water (S) conditions before the separation process. (b) Proteins from *N. oleoabundans* cultivated in fresh water recovered in the interface after the separation process using 1, PEG 400‐citrate; 2, Iolilyte 221PG‐citrate and 3, PEG 400‐Ch DHp.

In order to study the conformation of proteins before and after the separation process, Native‐PAGE electrophoresis was performed to the initial microalgae added and to the proteins recovered in the interface. Figure 5(a) shows the proteins in the extract of microalgae cultivated under saline and freshwater conditions. Rubisco (∼560 kDa) was identified as the most abundant protein known in *N. oleoabundans* and was therefore used as biomarker.^59^ Other proteins present in the extract have not been characterized, but other bands were detected. In Fig. 5(b), a Rubisco band appears for the three systems, indicating that this protein remains intact also after interfacial partitioning with the ATPSs. However, the band of Rubisco in the Iolilyte 221PG‐citrate system is faint, indicating the influence that the Iolilyte221PG has on the protein. The effect of Iolilyte221PG on Rubisco was studied previously, showing that when increasing the amount of this IL, the protein loses its native conformation and forms aggregates.^14^, ^29^ PEG‐Ch DHp and PEG‐citrate seem to retain the native conformation not only of Rubisco, but also of other proteins from *N. oleoabundans* (Fig. 5). These systems are therefore considered milder for the separation of proteins. However, further characterization and techno‐functional evaluation of the proteins recovered is needed for their use in different industrial applications.

### Selectivity

Table 3 shows the selectivity results of ATPSs for the fractionation of pigments and proteins from microalgae extracts. Polymer–IL ATPSs shows higher selectivity between proteins and pigments due to the preference of the pigments for the top phase and of the proteins for the bottom phase. These results suggest that polymer–IL systems do not exclude molecules by salting out, but hydrogen bonding and molecular interactions between the protein and ATPS components allow the molecules to partition, allowing a high selectivity between protein and pigments. As discussed before, this depends on protein concentration as high protein concentrations will cause significant size exclusion effects (interfacial partitioning).

**Table 3:** Selectivity results of the separation pigments/proteins from *N. oleoabundans* extract

ATPS	*K*_pigment/protein_	
	Saline	Freshwater
PEG 400‐citrate	0.26	0.18
Iolilyte 221PG‐citrate	0.54	0.64
PEG 400‐Ch DHp	14.18	11.61

**Figure 6:**
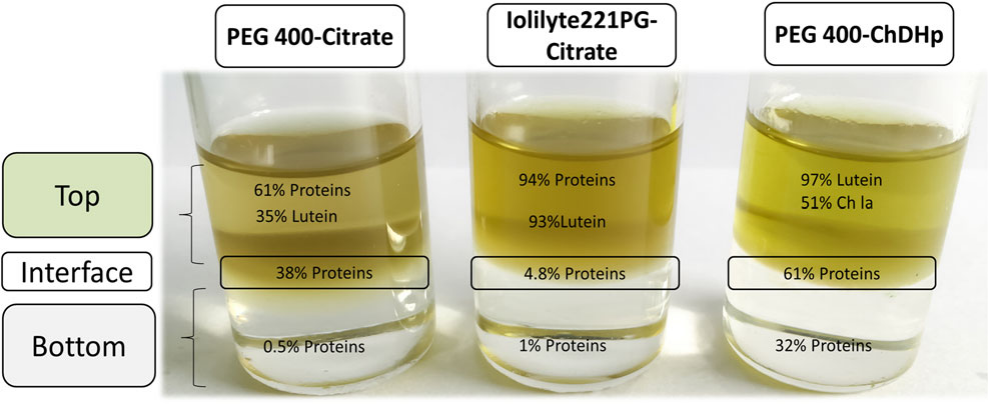
Summary of highest extraction efficiencies (%w/w) obtained in the separation of pigments and proteins from *N. oleoabundans* extract cultivated under saline conditions.

PEG400‐Ch DHp partition behaviour is clearly beneficial for the partitioning of the molecules in different phases (Fig. 6). Besides that, proteins recovered in the interface seem to conserve their native conformation based on electrophoresis experiments and pigments did not suffer oxidation in the top phase. Recovery of the proteins in the bottom phase and recycle of the cholinium dihydrogen phosphate for reuse is possible by ultrafiltration as described by Ramalho *et al*.^58^ A large number of biocompatible ILs such as Ch‐DHp are being synthetized,^60^ which opens up research opportunities for polymer–IL ATPSs. This type of ATPS and their recyclability will be studied in the future to separate microalgae biomolecules in an integrated biorefinery approach. The partitioning behaviour of other molecules of industrial interest such as lipids and carbohydrates from microalgae should be investigated to design a correct biorefinery approach.

## CONCLUSIONS

As a first step towards the development of a multiproduct microalgae biorefinery, three kind of ATPSs were investigated to separate proteins and pigments from microalgae extract. The traditional polymer–salt (PEG400‐citrate), and two IL‐ATPSs: IL–citrate (Iolilyte221PG‐citrate) and polymer–IL (PEG400‐Ch DHp). Although Iolilyte221PG‐citrate showed outstanding partition coefficient for pigments, a high amount of proteins also moved to the top phase. This behaviour resulted in low selectivity between pigments and proteins. The quality of pigments and proteins separated by Iolilyte 221PG‐citrate were considered low as pigments suffered degradation (oxidation) and the proteins did not retain their native form.

The PEG400‐Ch DHp system was the most selective for separating pigments (top) and proteins (interface and bottom): 97.3 ± 1.0% of the lutein content in *N. oleoabundans* extract was separated into the top phase and a very low amount of proteins moved to the top phase. Proteins moved preferentially to the bottom phase and interface. High protein concentration load resulted in interfacial precipitation, up to 92.2 ± 2.0% of proteins precipitated in the interface. This interfacial partitioning is considered an advantage in the separation of complex matrices such as microalgae, because it combines separation and concentration of proteins. Thus, proteins in the interface can be directly recovered and do not need to be extracted from the phase forming components. This simplifies the recycling of phase forming components. Besides that, proteins recovered in the interface seem to conserve their native conformation based on electrophoresis experiments and pigments did not suffer oxidation in the top phase. We demonstrated the potential of ATPS to separate different biomolecules simultaneously, giving value to different microalgae components for a sustainable multiproduct biorefinery. Furthermore, components from microalgae cultivated in saline water and freshwater can be selectively fractionated using ATPS. Using salt water to cultivate microalgae can reduce costs and the freshwater footprint for large scale production, which is an advantage in view of a sustainable process.

## Supporting information


**Table S1**. Composition of mixtures prepared for the partitioning of microalgae pigments and proteins.
**Table S2**. Effect of the TLL on the partitioning of pigments by the ATPSs. The results represent the average of two independent experiments with the respective standard deviations
**Table S3**. Effect of the TLL on the distribution of proteins among the three phases. The results represent the average of two independent experiments with the respective standard deviations
**Figure S1**. Effect of NaCl (%w/w) increase in the partitioning of pigments.) Iolilyte 221PG‐citrate;)PEG400‐citrate and) PEG400‐Ch DHp. Error bars indicate standard deviations. *Significant difference (*P* < 0.05).
**Figure S2**. Effect of ATPS components on pigments. (a) RP‐HPLC chromatogram of pigments present in saline microalgae; (b) RP‐HPLC chromatograms of pigments recovered in the top phase by ATPSs (450 nm).

## References

[1] Wijffels RH , Barbosa MJ and Eppink MHM , Microalgae for the production of bulk chemicals and biofuels. Biofuels Bioprod Biorefin 4:287–295 (2010).

[2] Ruiz J , Olivieri G , De Vree J , Bosma R , Willems P , Reith JH *et al*, Towards industrial products from microalgae. Energy Environ Sci 9:3036–3043 (2016).

[3] Eppink MHM , Olivieri G , Reith H , van den Berg C , Barbosa MJ and Wijffels RH , From Current Algae Products to Future Biorefinery Practices: A Review. Springer, Berlin, pp. 1–25 (2017). 10.1007/10_2016_64 28265702

[4] Mulders KJM , Lamers PP , Martens DE and Wijffels RH , Phototrophic pigment production with microalgae: biological constraints and opportunities. J Phycol 50:229–242 (2014). 26988181 10.1111/jpy.12173

[5] Patil G and Raghavarao KSMS , Aqueous two phase extraction for purification of C‐phycocyanin. Biochem Eng J 34:156–164 (2007).

[6] Castro‐Puyana M , Herrero M , Urreta I , Mendiola JA , Cifuentes A , Ibáñez E *et al*, Optimization of clean extraction methods to isolate carotenoids from the microalga Neochloris oleoabundans and subsequent chemical characterization using liquid chromatography tandem mass spectrometry. Anal Bioanal Chem 405:4607–4616 (2013). 23314588 10.1007/s00216-012-6687-y

[7] Shegokar R and Mitri K , Carotenoid lutein: a promising candidate for pharmaceutical and nutraceutical applications. J Diet Suppl 9:183–210 (2012). 22889143 10.3109/19390211.2012.708716

[8] Rasala BA and Mayfield SP , Photosynthetic biomanufacturing in green algae; production of recombinant proteins for industrial, nutritional, and medical uses. Photosynth Res 123:227–239 (2015). 24659086 10.1007/s11120-014-9994-7

[9] Becker EW , Micro‐algae as a source of protein. Biotechnol Adv 25:207–210 (2007). 17196357 10.1016/j.biotechadv.2006.11.002

[10] t Lam GP , Vermuë MH , Eppink MHM , Wijffels RH and van den Berg C , Multi‐product microalgae biorefineries: from concept towards reality. Trends Biotechnol 36:216–227 (2018). 29132753 10.1016/j.tibtech.2017.10.011

[11] Desai RK , Streefland M , Wijffels RH and Eppink MHM , Novel astaxanthin extraction from Haematococcus pluvialis using cell permeabilising ionic liquids. Green Chem 18:1261–1267 (2016).

[12] Chua ET and Schenk PM , A biorefinery for Nannochloropsis: induction, harvesting, and extraction of EPA‐rich oil and high‐value protein. Bioresour Technol 244:1416–1424 (2017). 28624245 10.1016/j.biortech.2017.05.124

[13] Cuellar‐Bermudez SP , Aguilar‐Hernandez I , Cardenas‐Chavez DL , Ornelas‐Soto N , Romero‐Ogawa MA and Parra‐Saldivar R , Extraction and purification of high‐value metabolites from microalgae: essential lipids, astaxanthin and phycobiliproteins. J Microbial Biotechnol 8:190–209 (2015). 10.1111/1751-7915.12167 PMC4353334 25223877

[14] Desai RK , Streefland M , Wijffels RH and Eppink MHM , Extraction and stability of selected proteins in ionic liquid based aqueous two phase systems. Green Chem 16:2670–2679 (2014).

[15] Zaslavsky BY , Bagirov TO , Borovskaya AA , Gulaeva ND , Miheeva LH , Mahmudov AU *et al*, Structure of water as a key factor of phase separation in aqueous mixtures of two nonionic polymers. Polymer 30:2104–2111 (1989).

[16] Rito‐Palomares M , The practical application of aqueous two‐phase processes for the recovery of biological products. J Microbiol Biotechnol 12:535–543 (2002).

[17] Pereira MM , Coutinho JAP and Freire MG , CHAPTER 8: ionic liquids as efficient tools for the purification of biomolecules and bioproducts from natural sources, in RSC Green Chemistry, ed. by Bogel‐Lukasik R Royal Society of Chemistry, London, pp. 227–257 (2016).

[18] Karunanithi AT and Mehrkesh A , Computer‐aided design of tailor‐made ionic liquids. AlChE J 59:4627–4640 (2013).

[19] Souza RL , Ventura SPM , Soares CMF , Coutinho JAP and Lima AS , Lipase purification using ionic liquids as adjuvants in aqueous two‐phase systems. Green Chem 17:3026–3034 (2015).

[20] Ventura SPM , Santos‐Ebinuma VC , Pereira JFB , Teixeira MFS , Pessoa A and Coutinho JAP , Isolation of natural red colorants from fermented broth using ionic liquid‐based aqueous two‐phase systems. J Ind Microbiol Biotechnol 40:507–516 (2013). 23455697 10.1007/s10295-013-1237-y

[21] Santos PL , Santos LNS , Ventura SPM , de Souza RL , Coutinho JAP , Soares CMF *et al*, Recovery of capsaicin from Capsicum frutescens by applying aqueous two‐phase systems based on acetonitrile and cholinium‐based ionic liquids. Chem Eng Res Des 112:103–112 (2016).

[22] Wang R , Chang Y , Tan Z and Li F , Applications of choline amino acid ionic liquid in extraction and separation of flavonoids and pectin from ponkan peels. Sep Sci Technol 51:1093–1102 (2016).

[23] Ventura SPM , e Silva FA , Quental MV , Mondal D , Freire MG and Coutinho JAP , Ionic‐liquid‐mediated extraction and separation processes for bioactive compounds: past, present, and future trends. Chem Rev 117:6984–7052 (2017). 28151648 10.1021/acs.chemrev.6b00550 PMC5447362

[24] Luo X , Smith P , Raston CL and Zhang W , Vortex fluidic device‐intensified aqueous two phase extraction of C‐phycocyanin from spirulina maxima. ACS Sustain Chem Eng 4:3905–3911 (2016).

[25] Benavides J and Rito‐Palomares M , Simplified two‐stage method to B‐phycoerythrin recovery from Porphyridium cruentum. J Chromatogr B Analyt Technol Biomed Life Sci 844:39–44 (2006). 10.1016/j.jchromb.2006.06.029 16860005

[26] Ruiz‐Ruiz F , Benavides J and Rito‐Palomares M , Scaling‐up of a B‐phycoerythrin production and purification bioprocess involving aqueous two‐phase systems: practical experiences. Process Biochem 48:738–745 (2013).

[27] Phong WN , Le CF , Show PL , Chang JS and Ling TC , Extractive disruption process integration using ultrasonication and an aqueous two‐phase system for protein recovery from Chlorella sorokiniana. Eng Life Sci 17:357–369 (2017). 10.1002/elsc.201600133 PMC6999311 32624781

[28] Gómez‐Loredo A , González‐Valdez J and Rito‐Palomares M , Insights on the downstream purification of fucoxanthin, a microalgal carotenoid, from an aqueous two‐phase system stream exploiting ultrafiltration. J Appl Phycol 27:1517–1523 (2015).

[29] Suarez Ruiz CA , van den Berg C , Wijffels RH and Eppink MHM , Rubisco separation using biocompatible aqueous two‐phase systems. Sep Purif Technol 196:254–261 (2017).

[30] Culture collection of algae and protozoa (2014) . Available: https://www.ccap.ac.uk/pdfrecipes.htm [August 2016].

[31] Bradford MM , A rapid and sensitive method for the quantitation of microgram quantities of protein utilizing the principle of protein‐dye binding. Anal Biochem 72:248–254 (1976). 942051 10.1016/0003-2697(76)90527-3

[32] Cisneros M , Benavides J , Brenes CH and Rito‐Palomares M , Recovery in aqueous two‐phase systems of lutein produced by the green microalga Chlorella protothecoides. J Chromatogr B Analyt Technol Biomed Life Sci 807:105–110 (2004). 10.1016/j.jchromb.2004.01.009 15177167

[33] Freire MG , Louros CLS , Rebelo LPN and Coutinho JAP , Aqueous biphasic systems composed of a water‐stable ionic liquid + carbohydrates and their applications. Green Chem 13:1536–1545 (2011).

[34] Montalvo‐Hernández B , Rito‐Palomares M and Benavides J , Recovery of crocins from saffron stigmas (Crocus sativus) in aqueous two‐phase systems. J Chromatogr A 1236:7–15 (2012). 22463999 10.1016/j.chroma.2012.03.012

[35] Quental MV , Caban M , Pereira MM , Stepnowski P , Coutinho JAP and Freire MG , Enhanced extraction of proteins using cholinium‐based ionic liquids as phase‐forming components of aqueous biphasic systems. Biotechnol J 10:1457–1466 (2015). 25864445 10.1002/biot.201500003

[36] Neves CMSS , Shahriari S , Lemus J , Pereira JFB , Freire MG and Coutinho JAP , Aqueous biphasic systems composed of ionic liquids and polypropylene glycol: insights into their liquid‐liquid demixing mechanisms. Phys Chem Chem Phys 18:20 571–20 582 (2016). 10.1039/c6cp04023c PMC5034898 27405841

[37] Thrane JE , Kyle M , Striebel M , Haande S , Grung M , Rohrlack T *et al*, Spectrophotometric analysis of pigments: a critical assessment of a high‐throughput method for analysis of algal pigment mixtures by spectral deconvolution. PLoS ONE 10:e0137645 (2015). 26359659 10.1371/journal.pone.0137645 PMC4567325

[38] Dilcher DL , Pavlick RJ and Mitchell J , Chlorophyll derivatives in middle Eocene sediments. Science 168:1447–1449 (1970). 17731591 10.1126/science.168.3938.1447

[39] Mantoura R and Llewellyn C , The rapid determination of algal chlorophyll and carotenoid pigments and their breakdown products in natural waters by reverse‐phase high‐performance liquid chromatography. Anal Chim Acta 151:297–314 (1983).

[40] Fernandes AS , Nogara GP , Menezes CR , Cichoski AJ , Mercadante AZ , Jacob‐Lopes E *et al*, Identification of chlorophyll molecules with peroxyl radical scavenger capacity in microalgae Phormidium autumnale using ultrasound‐assisted extraction. Food Res Int 99:1036–1041 (2017). 28865614 10.1016/j.foodres.2016.11.011

[41] Li T , Xu J , Wu H , Wang G , Dai S , Fan J *et al*, A saponification method for chlorophyll removal from microalgae biomass as oil feedstock. Mar Drugs 14:162 (2016). 10.3390/md14090162 PMC5039533 27618070

[42] Orr VCA , Plechkova NV , Seddon KR and Rehmann L , Disruption and wet extraction of the microalgae Chlorella vulgaris using room‐temperature ionic liquids. ACS Sustainable Chem Eng 4:591–600 (2016).

[43] Luechau F , Ling TC and Lyddiatt A , A descriptive model and methods for up‐scaled process routes for interfacial partition of bioparticles in aqueous two‐phase systems. Biochem Eng J 50:122–130 (2010).

[44] Kim CW , Interfacial condensation of biologicals in aqueous two‐phase systems. Doctoral dissertation, Massachusetts Institute of Technology, Department of Applied Biological Sciences (1987).

[45] Asenjo JA and Andrews BA , Aqueous two‐phase systems for protein separation: a perspective. J Chromatogr A 1218:8826–8835 (2011). 21752387 10.1016/j.chroma.2011.06.051

[46] Ketnawa S , Rungraeng N and Rawdkuen S , Phase partitioning for enzyme separation: an overview and recent applications. Int Food Res J 24:1–24 (2017).

[47] Chow YH , Yap YJ , Anuar MS , Tejo BA , Ariff A , Show PL *et al*, Interfacial partitioning behaviour of bovine serum albumin in polymer‐salt aqueous two‐phase system. J Chromatogr B: Analyt Technol Biomed Life Sci 934:71–78 (2013). 10.1016/j.jchromb.2013.06.034 23911538

[48] Matsuyama T , Domyoung K , Umetsu M , Ikawa T , Yamanishi M , Ishida N *et al*, Ionic liquid/water interfacial localization of a green fluorescent protein fused to a tryptophan‐rich peptide. J Biosci Bioeng 113:160–165 (2012). 22036073 10.1016/j.jbiosc.2011.09.016

[49] Alvarez‐Guerra E and Irabien A , Separation of proteins by ionic liquid‐based three‐phase partitioning, in Ionic Liquids in Separation Technology. Elsevier, Amsterdam, pp. 207–234 (2014). 10.1016/b978-0-444-63257-9.00006-7.

[50] e Silva FA , Caban M , Kholany M , Stepnowski P , Coutinho JAP and Ventura SPM , Recovery of nonsteroidal anti‐inflammatory drugs from wastes using ionic‐liquid‐based three‐phase partitioning systems. ACS Sustain Chem Eng 6:4574–4585 (2018).

[51] Alvarez‐Guerra E and Irabien A , Ionic liquid‐based three phase partitioning (ILTPP) for Lactoferrin recovery. Sep Sci Technol (Philadelphia) 49:957–965 (2014).

[52] Alvarez‐Guerra E and Irabien A , Ionic liquid‐based three phase partitioning (ILTPP) systems for whey protein recovery: ionic liquid selection. J Chem Technol Biotechnol 90:939–946 (2015).

[53] Jaeger LD , Carreres BM , Springer J , Schaap PJ , Eggink G , Martins Dos Santos VAP *et al*, Neochloris oleoabundans is worth its salt: transcriptomic analysis under salt and nitrogen stress. PLoS ONE 13:e0194834 (2018). 29652884 10.1371/journal.pone.0194834 PMC5898717

[54] Andrews BA and Asenjo JA , Protein partitioning equilibrium between the aqueous poly(ethylene glycol) and salt phases and the solid protein phase in poly(ethylene glycol)‐salt two‐phase systems. J Chromatogr B Biomed Appl 685:15–20 (1996). 8930748 10.1016/0378-4347(96)00134-x

[55] Ribeiro SC , Monteiro GA , Cabral JMS and Prazeres DMF , Isolation of plasmid DNA from cell lysates by aqueous two‐phase systems. Biotechnol Bioeng 78:376–384 (2002). 11948444 10.1002/bit.10227

[56] Amid M , Shuhaimi M , Islam Sarker MZ and Abdul Manap MY , Purification of serine protease from mango (Mangifera Indica Cv. Chokanan) peel using an alcohol/salt aqueous two phase system. Food Chem 132:1382–1386 (2012). 29243626 10.1016/j.foodchem.2011.11.125

[57] Li Z , Liu X , Pei Y , Wang J and He M , Design of environmentally friendly ionic liquid aqueous two‐phase systems for the efficient and high activity extraction of proteins. Green Chem 14:2941–2950 (2012).

[58] Ramalho CC , Neves CM , Quental MV , Coutinho JA and Freire MG , Separation of immunoglobulin G using aqueous biphasic systems composed of cholinium‐based ionic liquids and poly(propylene glycol). J Chem Technol Biotechnol 93:1931–1939 (2018). 10.1002/jctb.5594 PMC6161813 30270961

[59] Günerken E , D'Hondt E , Eppink M , Elst K and Wijffels R , Influence of nitrogen depletion in the growth of N. Oleoabundans on the release of cellular components after beadmilling. Bioresour Technol 214:89–95 (2016). 27128193 10.1016/j.biortech.2016.04.072

[60] Reid JESJ , Prydderch H , Spulak M , Shimizu S , Walker AJ and Gathergood N , Green profiling of aprotic versus protic ionic liquids: synthesis and microbial toxicity of analogous structures. Sustainable Chem Pharmacy 7:17–26 (2018).

