# Supplementary material for: Selective and mild fractionation of microalgal proteins and pigments using aqueous two‐phase systems
Source: J Chem Technol Biotechnol. 2018 Jul 3;93(9):2774–83. doi: 10.1002/jctb.5711 (PMC6099415; doi:10.1002/jctb.5711)
Supplement: Supplementary file 1 — Table S1. Composition of mixtures prepared for the partitioning of microalgae pigments and proteins. Table S2. Effect of the TLL on the partitioning of pigments by the ATPSs. The results represent the average of two independent experiments with the respective standard deviations Table S3. Effect of the TLL on the distribution of proteins among the three phases. The results represent the average of two independent experiments with the respective standard deviations Figure S1. Effect of NaCl (%w/w) increase in the partitioning of pigments.) Iolilyte 221PG‐citrate;)PEG400‐citrate and) PEG400‐Ch DHp. Error bars indicate standard deviations. *Significant difference (P < 0.05). Figure S2. Effect of ATPS components on pigments. (a) RP‐HPLC chromatogram of pigments present in saline microalgae; (b) RP‐HPLC chromatograms of pigments recovered in the top phase by ATPSs (450 nm). [file JCTB-93-2774-s001.docx]

Selective and mild fractionation of microalgal proteins and pigments using aqueous two phase systems.

Catalina A. Suarez Ruiz^a*^, Daniel P. Emmery^a^, Rene H. Wijffels^a, b^, Michel H.M. Eppink^a^, Corjan van den Berg^a^

^a^Bioprocess Engineering, AlgaePARC, Wageningen University, P.O. Box 16, 6700 AA Wageningen, The Netherlands

^b^Nord University, N-8049, Bodø, Norway

Corresponding author: Catalina Andrea Suarez Ruiz

Bioprocess Engineering Group
Wageningen University & Research
PO Box 16
6700 AA Wageningen
The Netherlands

Tel: +31 613903779

Email: [catalina.suarezruiz@wur.nl](mailto:catalina.suarezruiz@wur.nl)

**Supporting information**

Table 1S Composition of mixtures prepared for the partitioning of microalgae pigments and proteins.

| **PEG 400-Citrate** | |  |  |
| --- | --- | --- | --- |
| **Tie line** | **X_M_ (wt %)** | | **Y_M_(wt %)** |
| 1 | 31.292 | | 21.325 |
| 2 | 32.626 | | 21.851 |
| 3 | 35.723 | | 23.887 |
| 4 | 39.055 | | 25.192 |
| **Iolilyte 221PG-Citrate** | |  |  |
| 1 | 26.777 | | 18.742 |
| 2 | 28.871 | | 20.721 |
| 3 | 28.432 | | 25.348 |
| 4 | 31.257 | | 28.611 |
| **PEG 400-ChDHP** | |  |  |
| 1 | 30.413 | | 29.412 |
| 2 | 31.275 | | 33.301 |
| 3 | 35.016 | | 33.905 |
| 4 | 36.759 | | 35.813 |

Table 2S Effect of the tie line length on the partitioning of pigments by the ATPSs. The results represent the average of two independent experiments with the respective standard deviations

|  |  | **Fresh water** | **Saline** |
| --- | --- | --- | --- |
| **ATPS** | **TLL(w/w%)** | **K_p_** | **K_p_** |
| **PEG 400-Citrate** | 39.3 | 10.7±0.6 | 21.6±1.2 |
|  | 47.3 | 14.7±0.3 | 20.8±4.8 |
|  | 63.8 | 22.2±2.1 | 37.8±2.5 |
|  | 74.6 | 36.1±4.1 | 38.9±1.3 |
| **Iolilyte 221PG-Citrate** | 37.5 | 15.7±6.1 | 15.8±0.2 |
|  | 53.2 | 21.9±0.9 | 14.8±0.3 |
|  | 67.7 | 31.3±3.3 | 31.8±1.7 |
|  | 80.3 | 61.9±11.3 | 45.8±3.5 |
| **PEG 400-Ch DHp** | 53.1 | 1.1±0.02 | 2.2±0.0 |
|  | 72.9 | 2.2±0.3 | 4.8±0.3 |
|  | 85.3 | 3.7±0.5 | 2.8±1.0 |
|  | 93.3 | 7.2±0.7 | 7.0±0.8 |

Table 3S Effect of the tie line length (TLL) on the distribution of proteins among the three phases. The results represent the average of two independent experiments with the respective standard deviations

|  |  | **Extraction efficiencies (%w/w)** | | | | | | |
| --- | --- | --- | --- | --- | --- | --- | --- | --- |
|  |  | **Fresh Water** | | | **Salt** | | | |
| **ATPS** | **TLL** | **Bottom** | **Top** | **Interface** | | **Bottom** | **Top** | **Interface** |
| **PEG 400-Citrate** | 39.3 | 0.6 ± 0.1 | 36.70± 7.2 | 62.72± 7.3 | | 1.44± 0.2 | 70.06± 6.4 | 28.50± 6.7 |
|  | 47.3 | 0.3± 0.01 | 28.36± 0.8 | 71.31± 0.8 | | 0.92± 0.3 | 66.48± 5.3 | 32.59± 5.7 |
|  | 63.8 | 0.2± 0.02 | 24.47± 3.2 | 75.38± 3.2 | | 0.55± 0.1 | 61.37± 2.6 | 38.08± 2.5 |
|  | 74.6 | 0.1± 0.04 | 22.68± 5.4 | 77.20± 5.3 | | 0.52± 0.1 | 61.32± 0.9 | 38.16± 0.8 |
| **Iolilyte 221PG-Citrate** | 37.5 | 1.6± 0.4 | 52.53± 2.7 | 45.89± 3.1 | | 1.40± 0.4 | 90.73± 3.4 | 7.87± 3.8 |
|  | 53.2 | 1.3± 0.4 | 51.64± 10.2 | 47.02± 10.7 | | 1.08± 0.2 | 91.26± 3.7 | 7.67± 3.9 |
|  | 67.7 | 0.9± 0.1 | 42.41± 5.7 | 56.68± 5.8 | | 0.81± 0.4 | 86.93± 4.0 | 12.26± 4.4 |
|  | 80.3 | 0.4± 0.2 | 40.37± 8.0 | 59.22± 8.2 | | 0.96± 0.3 | 94.25± 2.8 | 4.79± 2.6 |
| **PEG 400-Ch DHp** | 53.1 | 11.5± 2.3 | 5.78± 2.0 | 82.68± 4.4 | | 43.07± 5.3 | 12.52± 1.8 | 44.41± 14.0 |
|  | 72.9 | 7.5± 0.7 | 4.36± 0.9 | 88.14± 0.3 | | 34.13± 0.5 | 10.62± 0.1 | 55.25± 0.4 |
|  | 85.3 | 5.3± 0.5 | 4.23± 1.0 | 90.52 ± 1.5 | | 24.87± 1.7 | 10.81± 0.8 | 64.32± 2.4 |
|  | 93.3 | 4.7± 1.0 | 3.08± 0.9 | 92.23± 1.9 | | 32.21± 0.7 | 6.73± 0.5 | 61.06± 1.2 |

**Figures**

*

Figure 1S Effect of NaCl (%w/w) increase in the partitioning of pigments. ) Iolilyte 221PG-citrate; )PEG400-Citrate and ) PEG400-Ch DHp. Error bars indicate standard deviations. *Significant difference (p < 0.05).


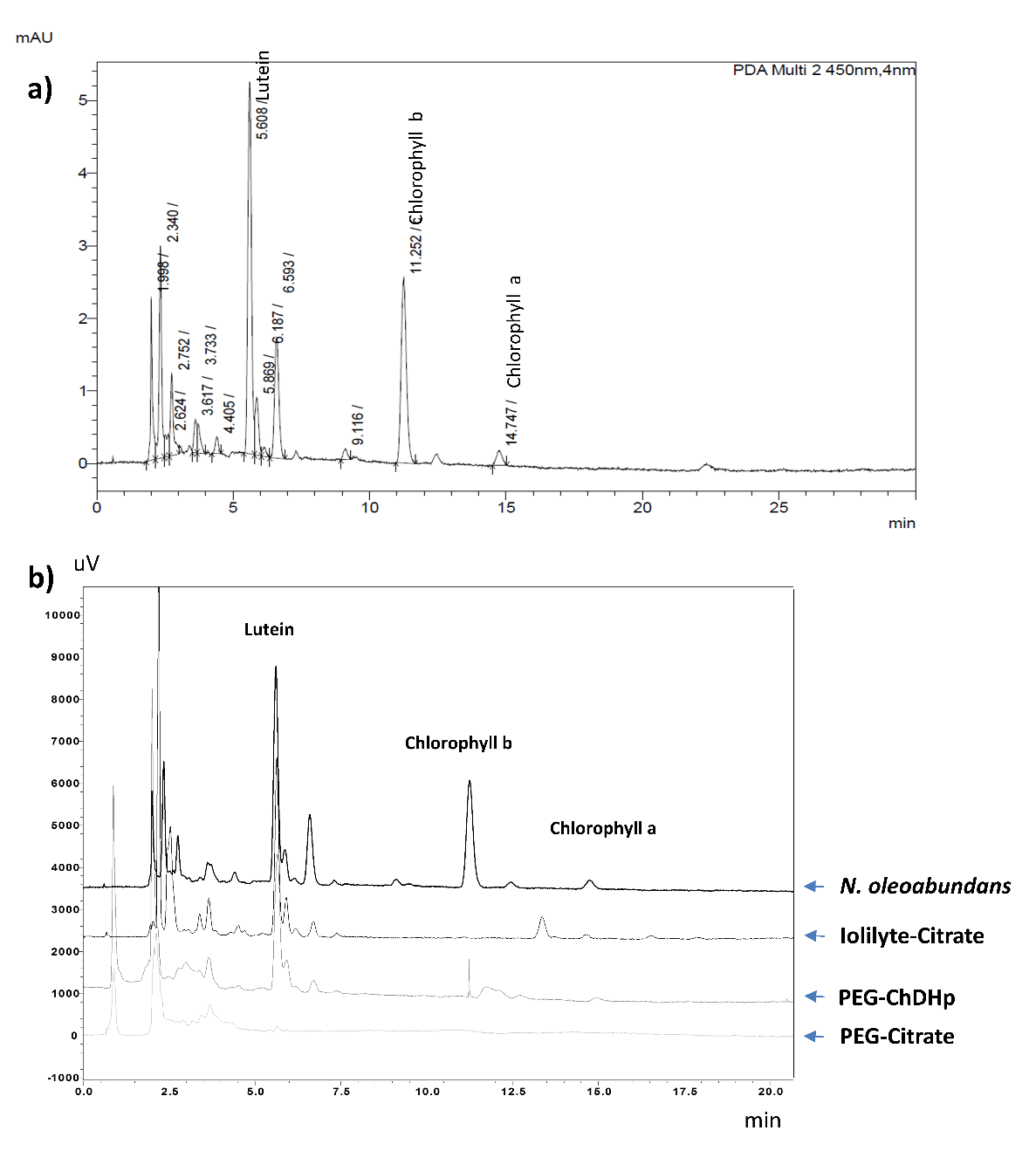


**Figure 2S** Effect of ATPS components on pigments. a) RP-HPLC chromatogram of pigments present in saline microalgae; b) RP-HPLC chromatograms of pigments recovered in the top phase by ATPSs (450 nm).
